# Assessing spatially explicit long-term landscape dynamics based on automated production of land category layers from Danish late nineteenth-century topographic maps in comparison with contemporary maps

**DOI:** 10.1007/s10661-025-13634-1

**Published:** 2025-01-25

**Authors:** Gregor Levin, Geoff Groom, Stig Roar Svenningsen

**Affiliations:** 1 Department of Environmental Science, Aarhus University, Frederiksborgvej 399, 4000 Roskilde, Denmark; 2 Department of Agroecology, Aarhus University, C. F. Møllers Allé 8, Bygning 1110, 8000 Aarhus C, Denmark; 3 Royal Danish Library, Special Collections, Søren Kierkegaards Plads. 1, 1221 Copenhagen K, Denmark

**Keywords:** Historical maps, Land use and land cover, Digital image processing, Automated production

## Abstract

Historical topographical maps contain valuable, spatially and thematically detailed information about past landscapes. Yet, for analyses of landscape dynamics through geographical information systems, it is necessary to “unlock” this information via map processing. For two study areas in northern and central Jutland, Denmark, we apply object-based image analysis, vector GIS, colour image segmentation, and machine learning processes to produce machine-readable layers for the land use and land cover categories forest, wetland, heath, dune sand, and water bodies from topographic maps from the late nineteenth century. Obtained overall accuracy was 92.3%. A comparison with a contemporary map revealed spatially explicit landscape dynamics dominated by transitions from heath and wetland to agriculture and forest and from heath and dune sand to forest. However, dune sand was also characterised by more complex transitions to heath and dry grassland, which can be related to active prevention of sand drift but that can also be biased by different categorisations of dune sand between the historical and contemporary data. We conclude that automated production of machine-readable layers of land use and land cover categories from historical topographical maps offers a resource-efficient alternative to manual vectorisation and is particularly useful for spatially explicit assessments of long-term landscape dynamics. Our results also underline that an understanding of mapped categories in both historical and contemporary maps is critical to the interpretation of landscape dynamics.

**Graphical abstract:**

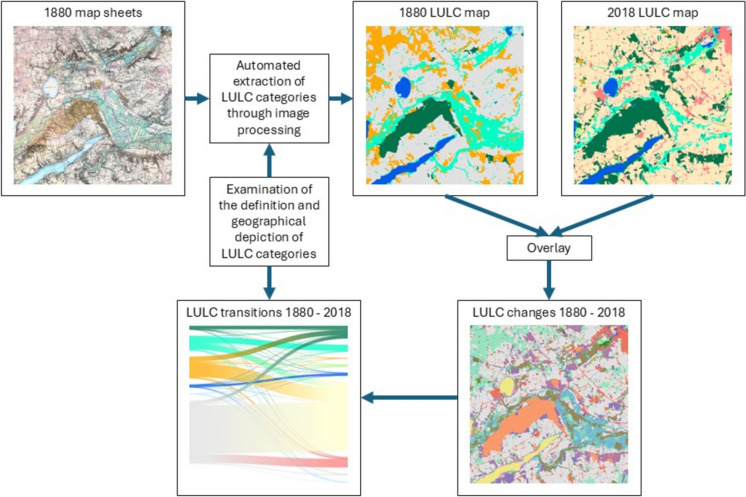

**Supplementary Information:**

The online version contains supplementary material available at 10.1007/s10661-025-13634-1.

## Introduction

Long-term, spatially explicit land use and land cover (LULC) information is critical to several applications, such as analyses of landscape dynamics and effect of changing drivers (Abadie et al., 2018; Konkoly-Gyuró et al., 2019; Levin & Kark, 2023; Mikusinska et al., 2013; Poska et al., 2018; Svenningsen et al., 2019; Van den Berghe et al., 2019). Long-term habitat continuity has positive effects on biodiversity (Hanski, 1999) as shown for, e.g. forest (Bradshaw et al., 2015; Joks et al., 2023; Kaim et al., 2016), grassland (Horstmann et al., 2023; Noda et al., 2022; Scherreiks et al., 2022), and scrubland (Gamboa-Badilla et al., 2020) habitats. Furthermore, LULC history highly affects greenhouse gas emissions and storage (Fuchs et al., 2016; Nitsch et al., 2018) and ecosystem service provision in general (Watson et al., 2021).

## The relevance of historical maps

Historical maps contain information about places, human activities, and biophysical properties in the past, for which no or only limited alternative spatial information sources exist (Chiang et al., 2016; Liu et al., 2019). In particular, topographic historical maps are among the most reliable representations of landscapes and LULC for the time prior to the existence of aerial photography and satellite imagery (Kaim et al., 2014; Loran et al., 2018). In topographic maps, categorisations of LULC are spatially explicit, i.e. are delineated as objects with specific locations and extents which can be applied in analyses of the presence, pattern, and distribution of LULC (Auffret et al., 2017; Mäyrä et al., 2023) and by comparison between historical and contemporary maps, LULC dynamics can be explored (Bürgi et al., 2017; Gobbi et al., 2019; Levin & Kark, 2023; Liu et al., 2018). Yet, historical maps are rarely complete, and definitions of LULC categories have changed over time and between different mapping regimes. E.g. Erikson and Skånes (2010) stress the importance of addressing inconsistencies in land cover definitions between different historical maps and for Danish wetlands, and Svenningsen et al. (2015) show a change from a military oriented mapping regime in historical maps to an environmental regime in modern maps.

## Digital map processing

In historical maps, information is expressed in colours, symbols, lines, and text labels. To make this information usable in a GIS, it must be “unlocked” through recognition and extraction via map processing. Traditionally, this process has involved time- and resource-intensive visual interpretation and manual delineation and applications were therefore often limited to relatively small study areas (Caspersen & Fritzbøger, 2002; Kienast, 1993; Kristensen et al., 2009; Mikusinska et al., 2013; Münier, 2009). Digital map processing refers to computational procedures aimed at automatically or semi-automatically creating digital spatial datasets of geographic features depicted in maps (Chiang et al., 2016; Freeman & Pieroni, 1982; Liu et al., 2019). There are two primary methodological approaches. The first involves recognising map symbols, linear features, and text labels by leveraging semantic knowledge associated with these. Here, specific rules are applied to extract these elements from the map data (Baily et al., 2011; le Riche, 2020; Liu et al., 2018; Pezeshk & Tutwiler, 2011; Uhl et al., 2018; Xu et al., 2016). Additionally, certain features, such as text items, are isolated and removed to enhance the extraction of the elements that are in focus (Gobbi et al., 2019; Kim et al., 2014; Mäyrä et al., 2023; Ostafin et al., 2017; Schlegel, 2023). The second approach, colour image segmentation (CIS), uses colours (e.g. blue for water, green for vegetation) to isolate specific geographic content. CIS employs various colour characteristics like RGB, hue, saturation, intensity, and grey scale transformations (Leyk & Llados, 2010). Depending on map complexity and quality, digital map processing has applied supervised classifications with training data to label image pixels or objects (Levin & Kark, 2023), unsupervised classifications without training data (Godfrey & Eveleth, 2015), or combined approaches (Liu et al., 2018).

Applications of digital map processing have been conducted for many historical maps and purposes. For Swedish district economic maps from the period from 1859 to 1934 and Swedish economic maps from the period from 1935 to 1978, Auffret et al. (2017) applied supervised image processing, based on RGB colour tones to extract arable land, forest, meadow, water, and open land. Baily et al. (2011) applied supervised classification techniques based on colour spaces, texture, and symbol patterns to extract seven land use categories from maps from the First Land Utilisation Survey of Britain from the 1930s. For Italian heritage maps from 1859, 1926, and 1992, Gobbi et al. (2019) applied object-based image analysis (OBIA) for filtering of undesirable map elements, such as text, symbols, and boundary lines and then used CIS to delineate LULC categories. Ostafin et al. (2017) applied supervised CIS and subsequent morphological techniques to filter out noise originating from undesired map elements to create a forest cover mask from historical topographic maps from Poland and Switzerland. For a region near Chancellorsville, USA, Liu et al. (2018) applied a pixel-based maximum likelihood classification (MLC) and an OBIA classification approach to a US civil war map and modern aerial photos to assess landscape dynamics from 1867 to 2014. Applying a supervised MLC and morphological operators to filter text items, Fuchs et al. (2015) elaborated a forest mask of central Europe based on a coarse scale land cover map for around 1900. Leyk and Boesch (2009) applied a combination of symbol recognition techniques and morphological operators to extract forest areas from Swiss, nineteenth-century topographic maps. For a study of 250 years of landscape change for Norfolk Island, Levin and Kark (2023) applied supervised classification and manual digitisation to extract detailed information on vegetation cover from 10 historical maps and datasets. Mäyrä et al. (2023) applied supervised CIS to historical maps to assess LULC changes from 1965 to 2022 with focus on agricultural land, mires, roads, water bodies, watercourses, and roads. More recent methodological developments include deep learning and convolutional neural network, e.g. for segmenting LULC categories from an eighteenth-century map of the Rhone basin (Martinez et al., 2023) for extracting wetlands from a 100-year-old historical map of Jönköping County in Sweden (Ståhl & Weimann, 2022) and for segmentation of wetland symbols from nineteenth-century maps from Ireland (O’Hara et al., 2024).

## The Danish perspective

Denmark has a long tradition for studies of land use and land cover change (LULCC) and landscape dynamics. However, besides a few studies based on statistical data (Frederiksen et al., 2009; Levin & Normander, 2008), most existing Danish work on historical landscape dynamics is limited to case studies at local or regional level (Caspersen & Fritzbøger, 2002; Jensen, 1964; Jensen & Jensen, 1977, 1979; Kristensen et al., 2009; Münier, 2009). The only nationwide study based on historical spatial data has been made on manually vectorised LULC data from an eighteenth-century topographic map from the Royal Danish Academy of Sciences and Letters (Dam, 2005; Korsgaard, 2004). However, the rather small scale of the original map (1:120,000) means that the data is not suitable for detailed analyses of landscape dynamics (Dam, 2008).

## Research gaps

Despite their relevance and numerous applications, there remain research gaps in relation to the use of historical maps, and particularly topographic maps. Several of the above-described examples of digital map processing focus on just one or a few LULC categories. Also, only few studies apply nineteenth-century topographic maps, which, compared to other historical maps, are very detailed and complex and thus more challenging for digital map processing. Finally, few studies deal with how changing definitions of LULC categories over time bias interpretation of LULC changes and landscape dynamics.

## Research questions

In this paper, we explore the technical possibility for extracting LULC data from Danish historical topographic maps from the late nineteenth century by automated methods and the applicability of this data in studies of LULCC. We focus on three research aims:

- Test the applicability of automated extracted machine-readable LULC categories from historical topographic maps for landscape change studies
- Explore landscape dynamic and spatially explicit change between different LULC categories
- Investigate how changes in categorisation of LULC between historical and contemporary maps, because of shifting mapping regimes, influence interpretation of changes in LULC and landscape dynamics

## Data and methods

### Study areas

**Fig. 1:**
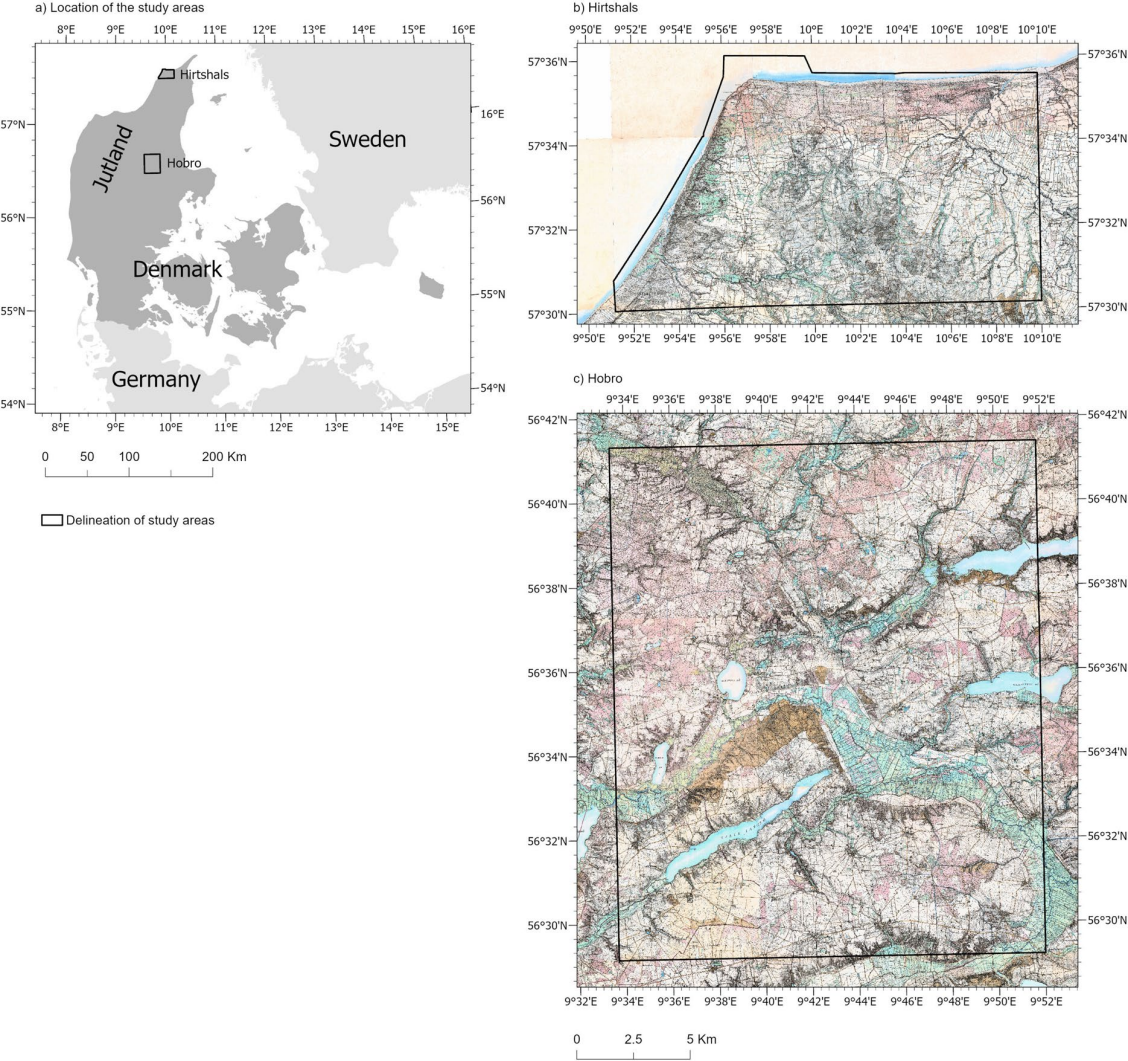
Location of the study areas (**a**), delineations of study areas overlaid on map sheets from the Høje Målebordsblade for Hirtshals (**b**), and Hobro (**c**)

Our study areas reflect two different Danish landscape types. The Hirtshals study area in northern Jutland (Fig. 1 b) covers 170 km^2^ and is characterised by aeolian sand along the coastline and late- and post-glacial marine sand and moraine clay inland. In the late 1800s, the area was dominated by vast areas of dune sand and heath along the coast and a mixture of agricultural land and wetland inland. The Hobro study area in mid-Jutland (Fig. 1 c) covers 425 km^2^ and is characterised by mixture of moraine sand and sandy clay and organic soils in river valleys. In the late 1800s, this area included extensive areas of heath, wetland, forest, and agricultural land.

### Applied maps

The first large-scale survey of the Danish landscape was done by the military in the second half of the nineteenth century, resulting in topographic maps in scale 1:20,000 published between 1870 and 1899 (hereafter referred to as “1880”). Although the landscape was surveyed from a military perspective (Svenningsen, 2016), these so called Høje Målebordsblade (HMB) maps are the most important source for detailed and spatially explicit information about the Danish landscape before the onset of industrial intensification of agriculture in the twentieth century. The HMB include detailed information about a range of LULC categories as well as point and linear features. However, to serve as a key, only a sample map with the range of different symbols was published but not including definitions of the different LULC categories and features, indicating that such information was deemed self-evident by the military surveyors at the time. Therefore, an archive-based historical study was conducted tracing the development of LULC categories back to the original survey instructions from the first half of the nineteenth century (Levin et al., 2020; Svenningsen et al., 2022). This led to the definition of a set of overarching LULC categories.

The HMB comprise approximately 1200 sheets and were published in two different versions, a black and white print, which was later hand-coloured, and a three-colour version (black, brown, and blue). While the three-coloured version relies on symbols for representing areal LULC categories, the hand-coloured version also utilises colours. For our study, we used two different versions of the hand-coloured maps: (i) a publicly available scanned and geo-rectified seamless raster dataset (SDFI, 2023) and (ii) the original hand-coloured paper map sheets, which are in general of higher quality in terms of the fidelity of line work and colour consistency. For our study areas, we acquired the original paper map sheets from the Map Collection at the Royal Danish Library (Royal Danish Library, 2024). We scanned those sheets at a resolution of 600 dpi, which is appropriate for 1:20,000 scale source material (Tobler, 1988) and georeferenced them using tie-points to the already georeferenced, raster data of the hand-coloured version, applying a spline model and nearest-neighbour resampling to 1-m cells.

Contemporary LULC information was derived from Basemap03, a nationwide LULC raster map for the year 2018 with a resolution of 10 × 10 m (Levin, 2019). We grouped the LULC categories from Basemap03 into nine major categories and categories from the HMB into six major categories (Table 1, Fig. S1, and Table S1 in the supplementary material). Categories for dry grassland and agriculture are only available in Basemap03 and we did not extract built/infrastructure features from the HMB maps. Yet, we incorporated these categories from Basemap03 as they can yield valuable insights into landscape dynamics.

**Table 1:** Description of employed LULC categories from applied historical and contemporary maps. The original classification systems and how these were aggregated into broader LULC categories for comparison in this study are presented in the supplementary material (Fig. S1 and Table S1)

	Høje Målebordsblade (HMB) (1880)	Basemap03 (2018)
Forest	Areas with tree cover. Includes deciduous and coniferous forest and young forest. Excludes orchards	Areas, dominated by woody vegetation. Includes temporary clear-cut areas. Excludes Christmas trees, energy forest, and orchards
Wetland	Areas with soft soil (often organic), and with potential for seasonal flooding. Vegetation cover includes permanent grass and natural vegetation. Includes wet meadows, bogs, and marshland	Uncultivated areas, dominated by grass and herb vegetation on permanently or seasonally waterlogged soils. Includes wet meadows, bogs, and marshland
Heath	Areas covered with heather vegetation (Heather or similar dwarf shrub)	Uncultivated areas, dominated by vegetation of heather or similar dwarf shrub
Dry grassland	Was not mapped as a category in the HMB	Grass and herb vegetation on dry and/or calcareous soils. Includes dunes covered with grass and herb vegetation
Dune sand	Areas characterised with loose soil in form of sand. Includes dunes and sand surfaces	Uncultivated areas, dominated by dune sand and coastal sand without or with sparse vegetation
Water body*	Permanent open water bodies. Includes water bodies covered by reed vegetation	Permanent open water bodies. Includes water bodies covered by reed vegetation
Agriculture	Was not mapped as a category in the HMB	Cropland and grassland for agricultural production, except grassland, which is also mapped as wetland, heath, or dry grassland
Built-up	Was not extracted from the HMB in this study	Built-up land, including buildings and transport infrastructure and associated land, such as gardens, parks, concrete surfaces, and road and rail verges
Other	Other LULC, including built-up land and transport infrastructure, which, in this study, was not extracted and agricultural land, which was not mapped as a category in the HMB	Other LULC, which is not included in any of the other LULC categories

*For the change assessment, water bodies were further divided into freshwater and sea

### Automated production of machine-readable layers of LULC categories

**Fig. 2:**
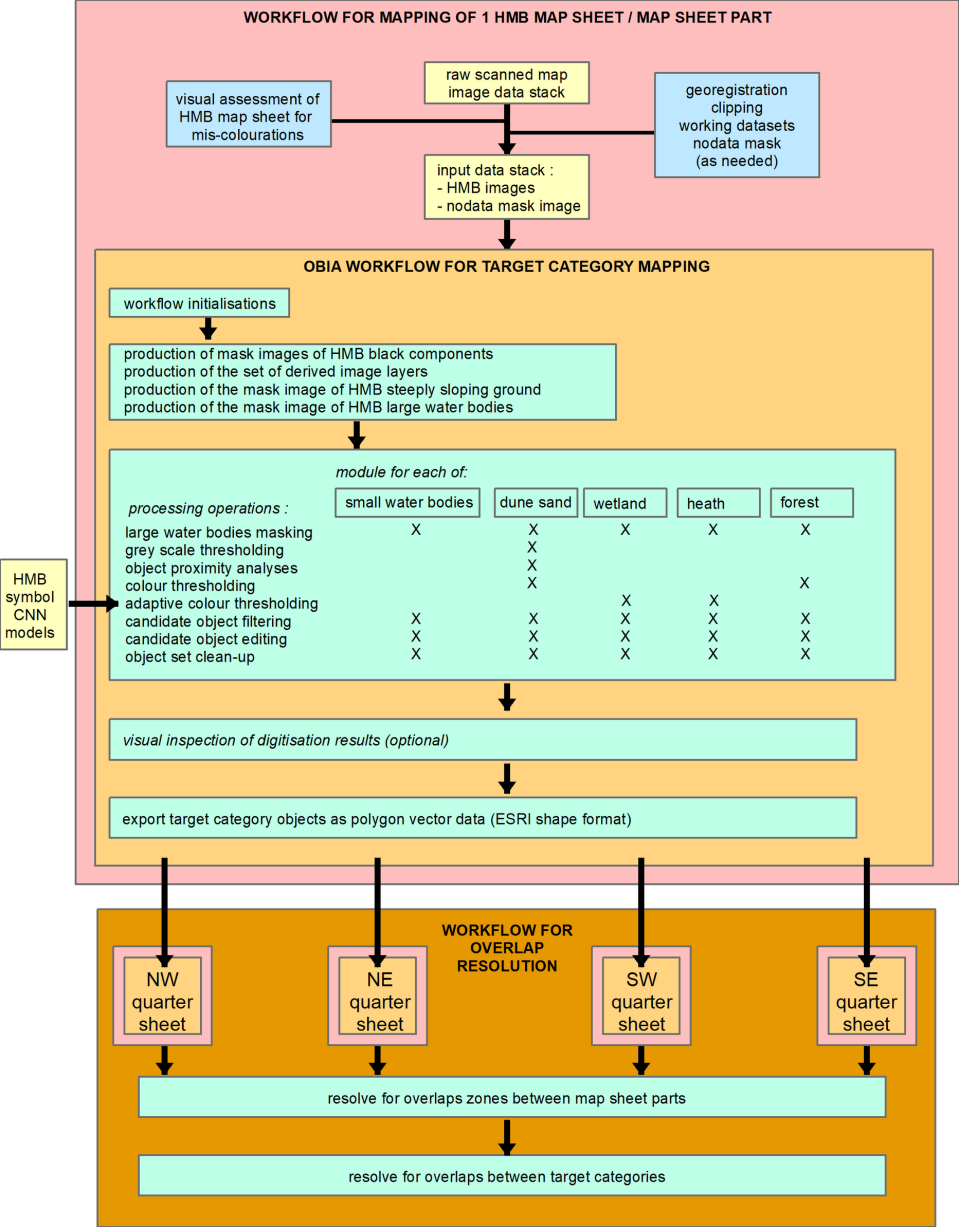
The workflow for the digital image processing of the HMB source material, comprising the modular LULC category mappings (pink box) workflow and the workflow for the integration of the mappings of the separate HMB quarter sheets (orange box)

Production of machine-readable vector geodata from the scanned historical maps (hereafter referred to as “the mapping”) was done as a set of modular sequences of raster, vector, and object processing procedures of a single commercial software (Trimble eCognition^©^ 9) that included image enhancement, CIS, OBIA, and machine learning (ML) methods. This section gives an overview, while detailed descriptions of the applied methods can be found in Groom et al. (2020) and Groom et al. (2021) and in Levin et al. (2020). Figure 2 presents an overview of the general workflow. We selected five target categories: heath, dune sand, wetland, forest, and water bodies as these are of relevance to issues of 100 + year LULCC and are extensively represented within the study areas. Modules and module sequences were customised for each of the five target categories and were developed to operate fully and without additional user input for all parts of both study areas. The mapping of each target category was made as an independent module and was also done independently for the extent of each HMB map sheet on account of inter-sheet differences, then cut into quarters (with 1 km E-W and N-S overlaps) on account of computer RAM considerations. In general, it was the Royal Danish Library sourced raster data that were used in the mapping, on account of the superior linework and colour quality; for some sheets however, colours in that material had faded, requiring instead use of raster data map sheet extracts from the seamless raster dataset (SDFI, 2023). Initial inspection was made of each sheet to check for the consistency of target category representations to the general HMB patterns and for any systematic HMB map quality issues, such as localised fading. If quality issues were noted, heuristic decisions were made related to (a) which of the two alternative HMB rasters to work with; (b) the need for use of a more customised, interactive setting of segmentation thresholds; and (c) the need for undertaking the mapping upon other partitioning of a map sheet.

**Fig. 3:**
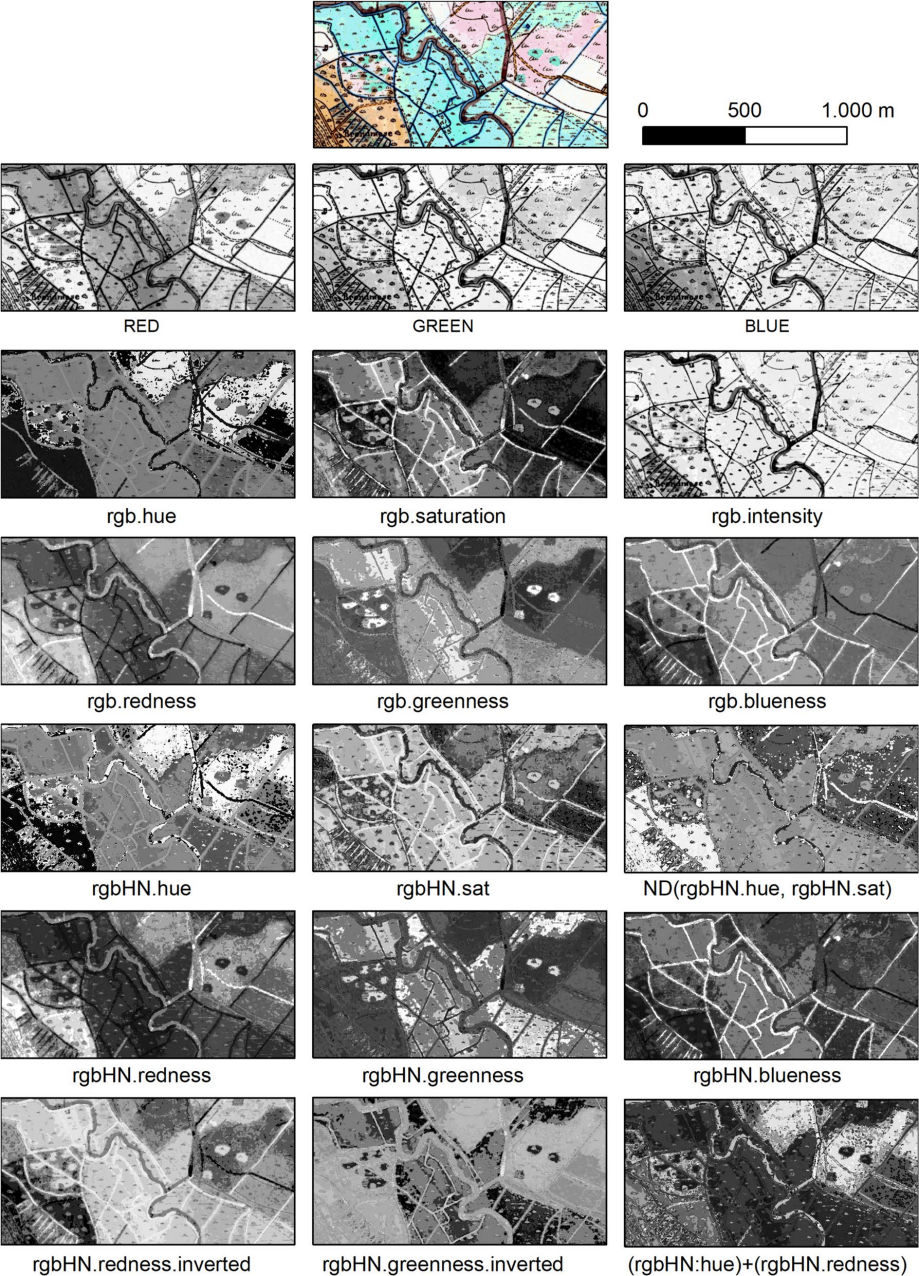
Illustration of the ability of a wide range of derived image layers to enhance different colour components of the HMB maps. The example used is a part of the seamless raster dataset of the Hobro study area. All parts are displayed with application of a 3.0 standard deviation stretch based on the statistics of the displayed data. HN, histogram normalised. ND, normalised difference. Note the lower degree of differences between HMB categories in the raw red, green, and blue image layers (second row from top) compared to the derived image layer (rows 3–7)

For the target categories, heath, wetland, forest, and water bodies occurrences in the HMB are characterised by use of colour. For these four, the applied mapping method was in general the same, consisting of determining which of a set of image enhancement rasters best isolated the target category, segmenting on a threshold in that raster and application of spatial object processes to improve upon the initial segmentation (Levin et al., 2020). Image enhancements to provide a set of derived rasters included hue, saturation, and intensity (HSI) transformations, and redness (R/(G + B)), greenness (G/(R + B)), and blueness (B/(R + G)) transformations applied upon raw, histogram normalisation and inverted forms of the image data. Figure 3 shows examples of these derived rasters, indicating how they enable isolation of the target categories. For smaller water bodies and for forest, the colour of the target category areas showed sufficient consistency across the map sheets to allow for application of a single (raster + threshold) rule for each. The colour of both wetland and heath in the applied HMB map sheets was far more variable, requiring a different approach (Groom et al., 2020), drawing upon the property that wetland and heath are each also associated with use of distinct respective symbols. In this, a machine learning (ML), convolutional neural network (CNN) was trained and then applied to detect occurrences of each symbol. From accurate ML detection of even just some of the symbols, it was possible to determine in an automated way, for each map sheet, the appropriate derived (raster + threshold) rule to apply as part of the general method.

The dune sand target category is distinct in that its representation is not associated by any use of areal colour hand-painting, but only by use of a black stippling (dots). The dune sand dot size and density are relatively constant, so having isolated all possible dune sand dots as black parts with an object size of just a few pixels, object-based distance and density methods and criteria were applied to form image object polygons of the extents of the dune sand (Groom et al., 2021).

As assists to the mapping of each of the target categories, masks were produced from the HMB and applied in the general method. A mask of all the black parts was generally applied for the post-segmentation processes, such as for filling parts of the target category’s extent where there was map text or linework (e.g. height contours). An assist mask was also formed from the HMB data of the postulated full extent of any large water bodies, both inland and off-coast (Levin et al., 2020); this was prescribed since these geographic features in the HMB often have highly variable colourations (e.g. large lakes are often hand coloured in blue only close to their shores). Furthermore, there is often marked bi-modality in the overall size range of “blue” water body extents (i.e. some very large plus many much smaller water bodies), which presented challenges for mapping of all water bodies with the general method. The mapping of water bodies in this work has not included mapping of rivers except where these widen to form lake-like features; the coincidence of many rivers in the HMB with administrative boundaries, which are presented as broad features in various colours, led to the decision that it was not possible to include rivers in the mapping of the water bodies target category in a consistent manner. The solid black lines that mark the limit of water surfaces were applied, via the mask of black parts, as an assist in the mapping of the water body target category, since there were cases were the blue hand-colouring was spread over parts outside the apparent HMB feature.

The HMB include delineation of the forest and heath categories on steeply sloping ground. Steeply sloping ground is itself designated by use of black lines crossing between the black height contours, a cartographic technique that we believe is unique to the HMB. That results in the colouration representations of forest and heath on steep ground being broken into extents comprising just a few image pixels, which would not be detected by the general methods for forest or heathland detection. The applied solution was to form a mask of steeply sloping ground from the mask of the black parts. The general methods for forest and heath were then applied within those mask extents with a far smaller minimum size threshold.

For improvement upon the initial target category object mappings, size thresholds were applied for filling small holes in target category extents and for removal of tiny cases of commission errors. Pixel-based object extent re-sizing methods were also applied including with control via surface tension values. The final mappings of target categories for the entireties of the two study areas was done by a second OBIA workflow (Fig. 2, lower part). A first part of this addressed inconsistencies, such as arising from the use of object size criteria, within the quarter-sheet overlap zones. Target category disjoints across map sheet borders, occasionally occurring locally where mappings from the two sets of source HMB material met, were addressed using a set of neighbourhood analysis based decision rules (Levin et al., 2020).

A key characteristic of the applied workflow for the automated production of machine-readable geodata is that it represents a toolbox of a set of tried-and-tested techniques that were then applied, in various combinations, for each of the five target categories. While the mapping was done, for each target category, by turnkey running of the workflow for that category, the more interactive visual inspections of each HMB raster dataset and the product of each mapping are nonetheless also considered as key steps of the overall workflow. The resulting 1 m raster data of the target LULC categories were also archived as un-smoothed vector polygon data.

### Accuracy assessment

A validation layer was created as a regular point grid with an equidistance of 100 m and a total of 27,498 validation points. The distance of 100 m was small enough to capture the variation of different LULC categories while being manageable to interpret within the resources of this project. Each point was classified by visual assessment based on the colours and signatures present up to 50 m from the point. The visual assessment was conducted by a student assistant. A point was classified either as a pure category, such as heath or wetland, or as a combination of several categories if these were present within 50 m from the point, such as heath and wetland or as heath and forest. The extracted polygon features were overlaid with the validation layer, and for each target category, proportions of false positive and false negative were calculated. In case of combinations of several categories, a point was assessed correctly classified if one of the assessed categories did match the extracted category.

### Assessing LULC dynamics

To map LULC dynamics from 1880 to 2018, we integrated LULC category layers derived from HMB map sheets with contemporary LULC data from Basemap03. The produced vector layers of HMB LULC categories were converted to 10 × 10 m raster corresponding to the resolution of Basemap03. To reduce the bias from small discrepancies between the delineations of LULC categories in the two maps, we spatially aligned the rasters of HMB categories to Basemap03 if they were located within 40 m from the same categories in Basemap03.

## Results

### Automated extraction of LULC categories from historical maps

**Fig. 4:**
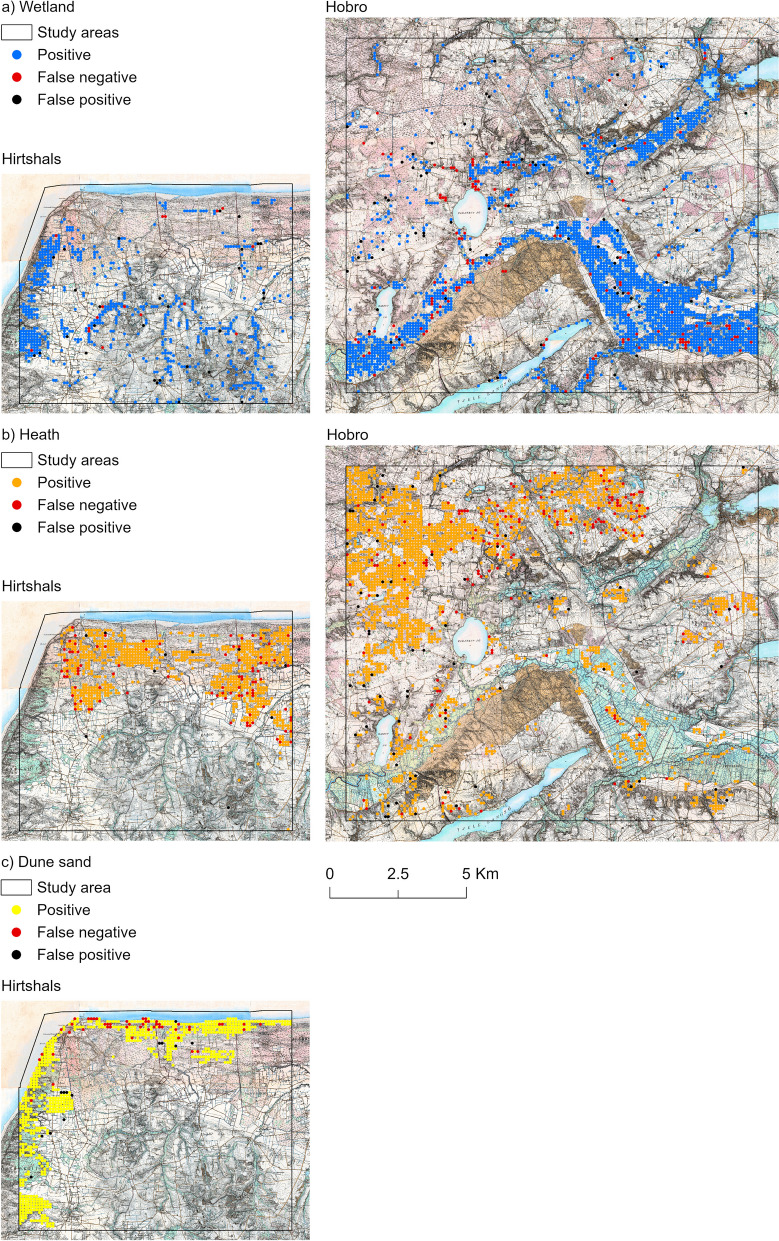
Validation points grouped into positives (matches), false negative, and false positive for both study areas and for the categories wetland (**a**), heath (**b**), and dune sand (**c**)

Table 2 summarises the accuracy assessment of the extracted LULC categories from the HMB. The overall achieved accuracy is 92.3%. All false-positive values are below 10%, indicating that our mapping does not significantly overestimate the area of the LULC categories. False-negative values exceed 10% for wetland, heath, and dune sand indicating that our mapping was less successful at consistently recognising these categories. For these categories, the maps in Fig. 4 show the validation points grouped into true positives (matches), false negatives, and false positives. For all three categories, the spatial distributions of false negatives show localised, semi-clustered patterns. This suggests special local factors leading to repeated occurrence of an error within a local area, such as narrow extents of a category (e.g. for wetland in a narrow-bottomed valley), markedly cut-up by text and linework. The few false-positive cases show dispersed patterns adjacent to positive cases, suggesting small delineation discrepancies in the produced mapping. Vast extents of the occurrences of each class are free of false negatives and false positives.

**Table 2:** Result of accuracy assessment for extracted LULC categories from the HMB

Extracted layers	Validation layers							
	Forest	Wetland	Heath	Dune sand	Water body	Other LULC	Total	False positive (%)
Forest	1159	2	3	0	0	18	1182	1.9
Wetland	3	2932	22	2	8	178	3144	6.7
Heath	2	15	4664	2	5	174	4860	4.0
Dune sand	0	3	4	726	7	15	753	3.7
Water body	0	14	2	9	1021	6	1052	2.9
Other LULC	63	596	761	142	55	14,893	16,508	9.8
Total	1227	3561	5455	880	1095	15,282	27,498	
False negative (%)	5.5	17.7	14.5	17.5	6.8	2.5

### Overall landscape dynamics

**Fig. 5:**
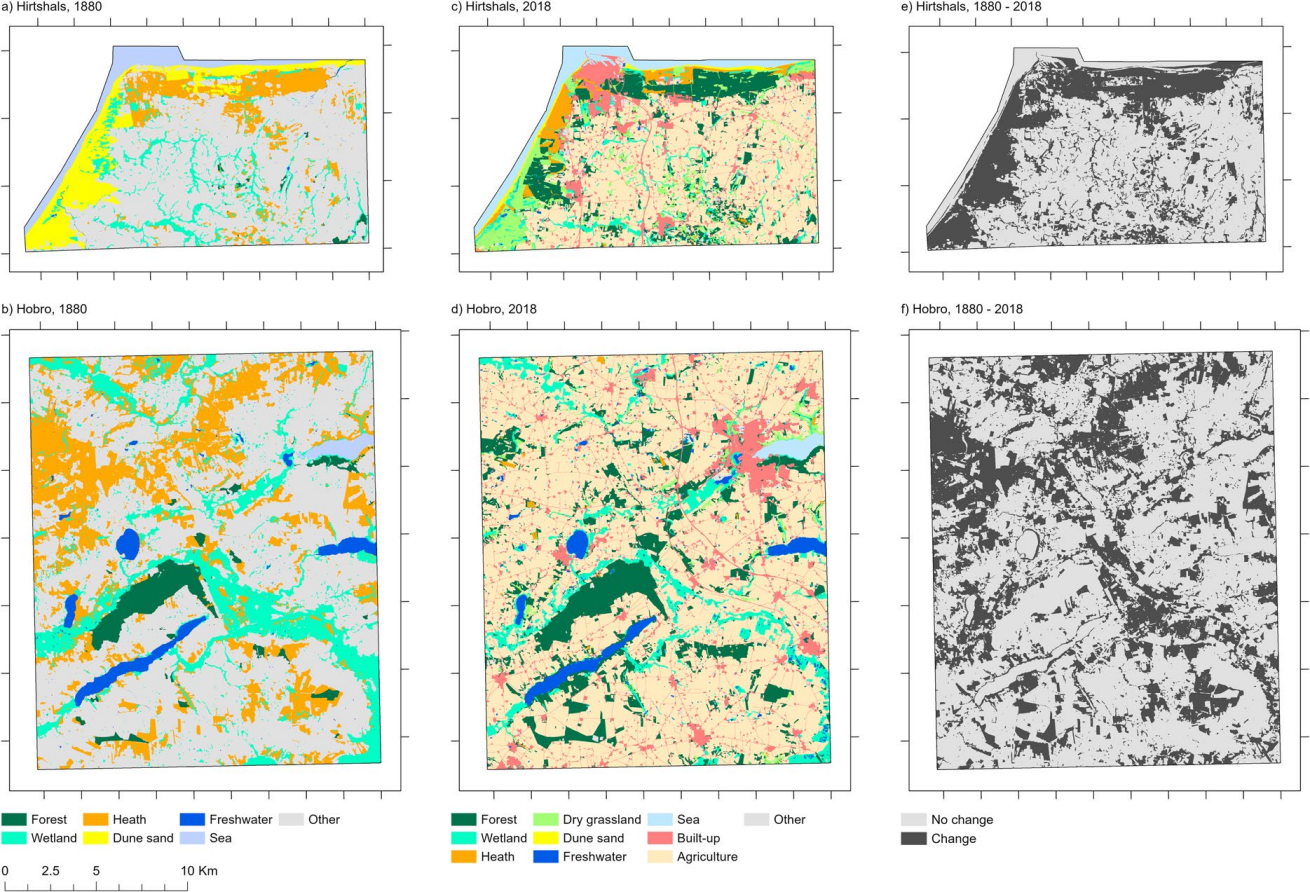
Mapped LULC categories for 1880 in Hirtshals (**a**) and Hobro (**b**); mapped LULC categories for 2018 in Hirtshals (**c**) and Hobro (**d**); changes between categories from 1880 to 2018 (not including changes from other) in Hirtshals (**e**) and Hobro (**f**)

**Fig. 6:**
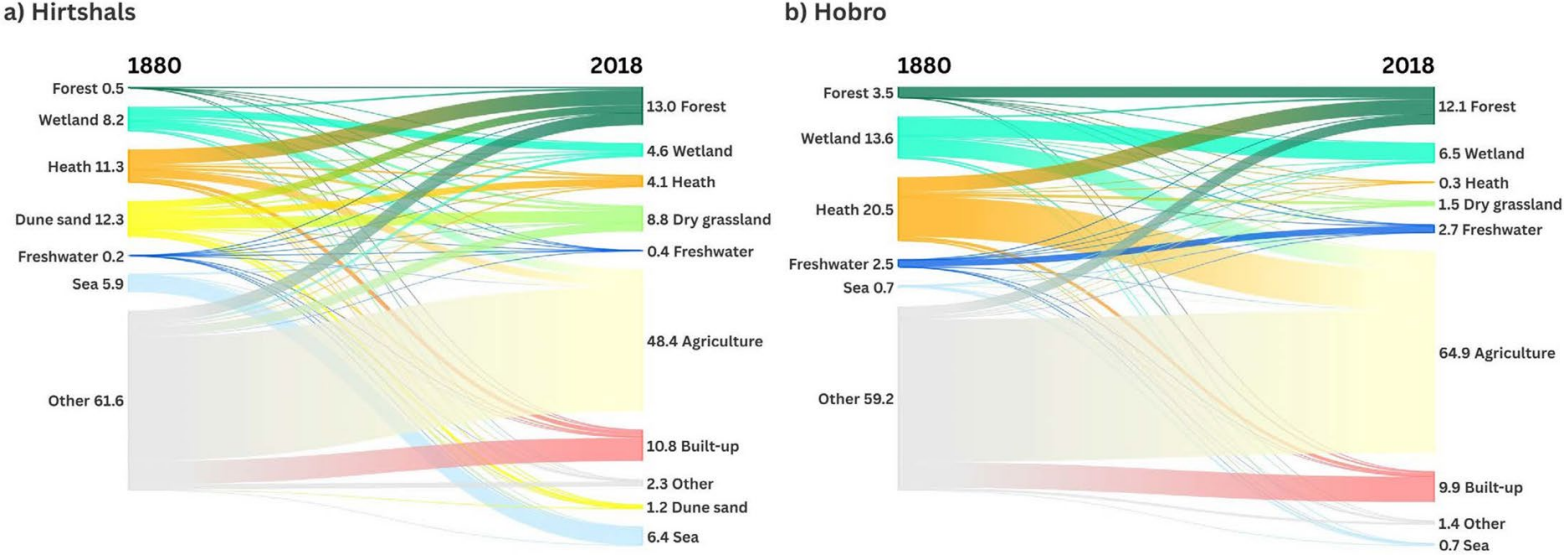
Spatially explicit changes between LULC categories from 1880 to 2018 in Hirtshals (**a**) Hobro (**b**). Values show proportion (%) of the whole study areas. Created in https://flourish.studio/

For the Hirtshals and the Hobro area, Fig. 5 shows the mapped LULC categories in 1880 and 2018 and areas, where categories have changed from 1880 to 2018. Figure 6 shows the proportion of LULC categories within each study area in 1880 and 2018 and spatially explicit transitions between LULC categories. The Hirtshals area is characterised by an increase in forest mainly at the expense of heath, dune sand, and other land and some transition from wetland and heath to agricultural land. Furthermore, a considerable area of dune sand also changed to heath and dry grassland. The Hobro area is characterised by afforestation, mainly at the expense of heath and other land and transitions from wetland and heath to agricultural land.

### Spatially explicit dynamics for selected LULC categories

**Fig. 7:**
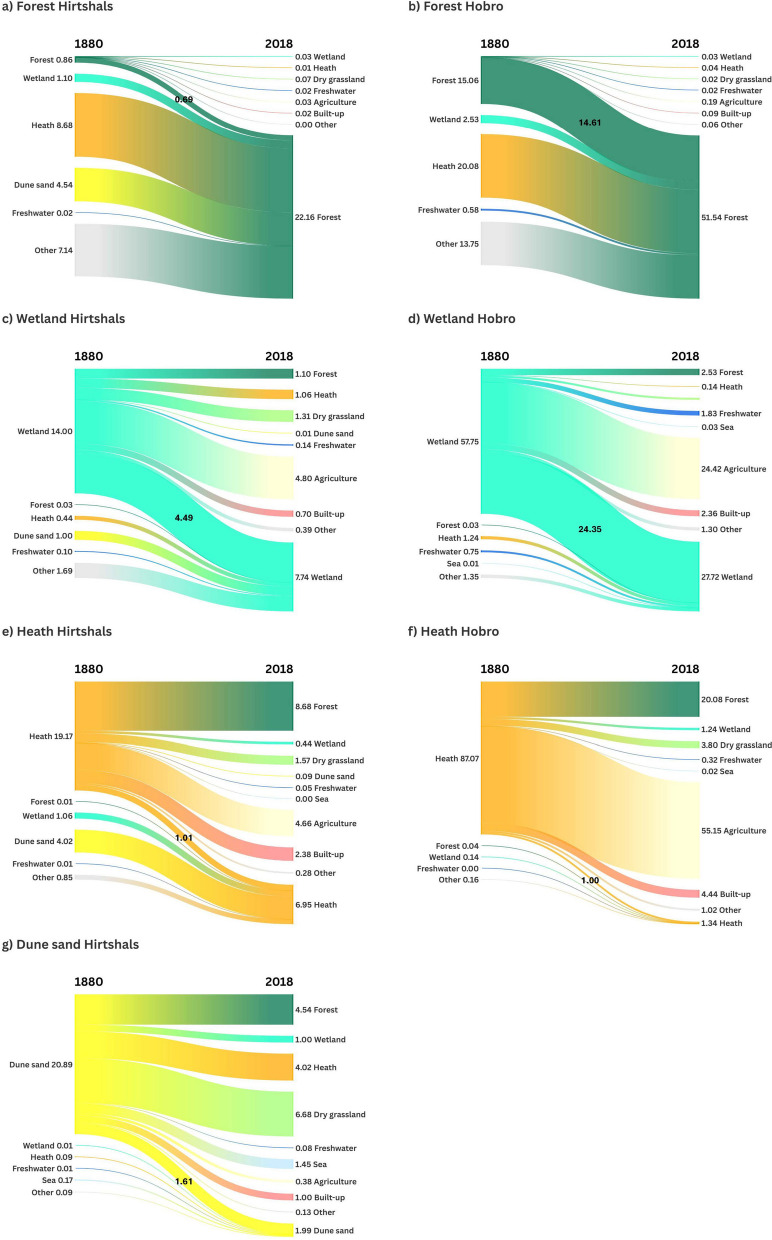
Spatially explicit changes from and to selected LULC categories from 1880 to 2018 for forest in Hirtshals (**a**) and in Hobro (**b**), Wetland in Hirtshals (**c**) and in Hobro (**d**), Heath in Hirtshals (**e**) and in Horbro (**f**), Dune sand in Hirtshals (**g**). Values show area in square kilometres. Created in https://flourish.studio/

**Fig. 8:**
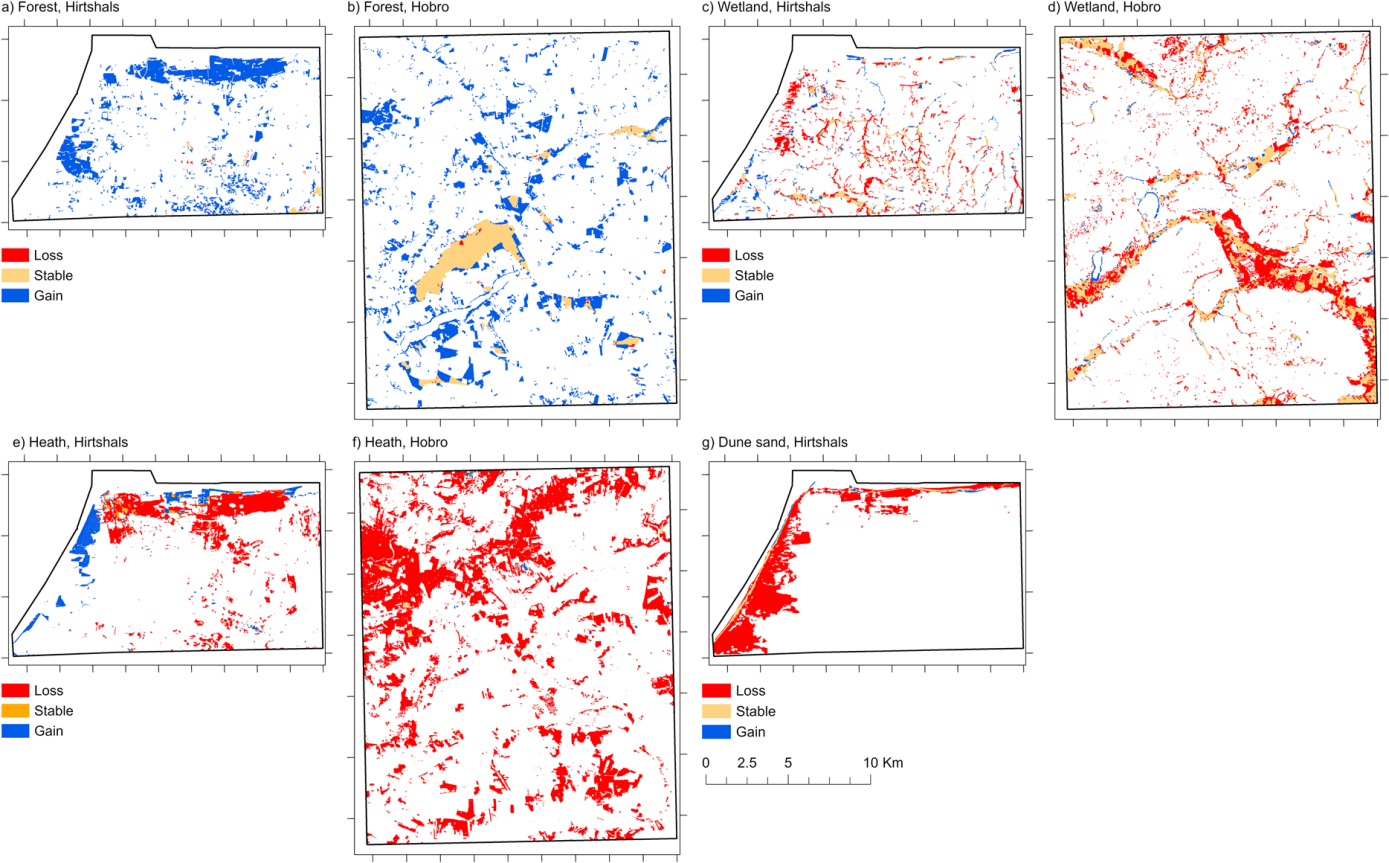
Spatial distribution of lost, stable, and gained areas of selected LULC categories from 1880 to 2018 for forest in Hirtshals (**a**) and in Hobro (**b**), Wetland in Hirtshals (**c**) and in Hobro (**d**), Heath in Hirtshals (**e**) and in Horbro (**f**), Dune sand in Hirtshals (**g**)

To illustrate the potential of detailed spatially explicit mapping of LULC categories from historical and contemporary sources, in the following paragraphs, we present spatially explicit dynamics for selected LULC categories. For the categories forest, wetland, heath, and dune sand, the diagrams in Fig. 7 show spatially explicit changes or transitions. Figure 8 shows spatial distributions of lost, stable, and gained areas for these categories.

#### Forest

Both study areas are characterised by a noticeable increase in forest area at the expense of open nature types (heath, wetland, dune sand) and other land, which includes agriculture (Fig. 7a and b). While in Hobro around 28% of the 2018 forest area displayed stability (Fig. 7b), this was only the case for around 3% of the 2018 forest area in Hirtshals (Fig. 7a). In both study areas, a negligible area of forest changed to other categories. While in Hirtshals, gained forest concentrates along the coast in the northern and western part of the study area (Fig. 8a), and gained forest is more scattered in Hobro (Fig. 8b).

#### Wetland

In both study areas, a considerable area and proportion of wetland was converted to agriculture. Around 34% of the 1880 wetland area in Hirtshals (Fig. 7c) and around 42% of the 1880 wetland area in Hobro (Fig. 7d) were in 2018 mapped as agriculture. While in Hobro the area, which changed from other land categories to wetland, is small, in Hirtshals, around 12% of the 2018 wetland area was not mapped as wetland in 1880. In both study areas, a considerable proportion of wetland was stable with around 32% of the 1880 wetland area in Hirtshals and around 42% of the 1880 wetland area in Hobro. Stable areas of wetland are concentrated in low lying areas and along watercourses (Fig. 8c and d).

#### Heath

In both study areas, a considerable area and proportion of heath was converted to forest and to agriculture. In Hirtshals, around 45% of the 1880 heath area was in 2018 mapped as forest and around 23% as agriculture (Fig. 7e). In Hobro, around 63% of the 1880 heath area was in 2018 mapped as forest and around 24% as agriculture (Fig. 7f). While in Hobro the area that changed from other LULC categories to heath is small, in Hirtshals, around 20% of the 2018 heath area was mapped as other categories in 1880, particularly dune sand. In both study areas, only a small proportion of heath was stable with around 4% of the 1880 heath area in Hirtshals and around 1% in Hobro. In Hirtshals, around 58% of the 2018 heath area was in 1880 mapped as dune sand. In both areas, stable areas of heath are characterised as small patches (Fig. 8e and f). In Hirtshals, gained areas of heath are concentrated along the coast in the western and northern part of the study area (Fig. 8e).

#### Dune sand

In Hirtshals, around 22% of the 1880 dune sand area was in 2018 converted to forest (Fig. 7g). Furthermore, around 19% was in 2018 mapped heath and around 32% as dry grassland. It can also be noted that around 7% of the 1880 dune sand area by 2018 was lost to sea. Stable dune sand areas are mainly found along the coastline (Fig. 8g).

## Discussion

Our results are in line with the overall landscape dynamics found in other Danish studies (Caspersen & Fritzbøger, 2002; Jensen, 1964; Jensen & Jensen, 1977, 1979; Kristensen et al., 2009; Münier, 2009), which document a general trend of afforestation and expansion of agriculture at the expense of open nature, such as wetland, heath, and dune sand. This development has led to a loss of and fragmentation of these habitat types and to a more homogeneous landscape, dominated by agriculture and forest. Furthermore, spatially explicit information with high spatial resolution allows identification of loss, stable, and gain of LULC categories, which is critical information to nature protection and restoration, climate change mitigation, and climate adaptation. However, in such studies, there is fundamental need for detailed investigations and analyses of potential difference in categories between the historical and contemporary map data to distinguish real changes from changes due to changes in mapping practices between historical and modern maps (Svenningsen et al., 2015).

**Fig. 9:**
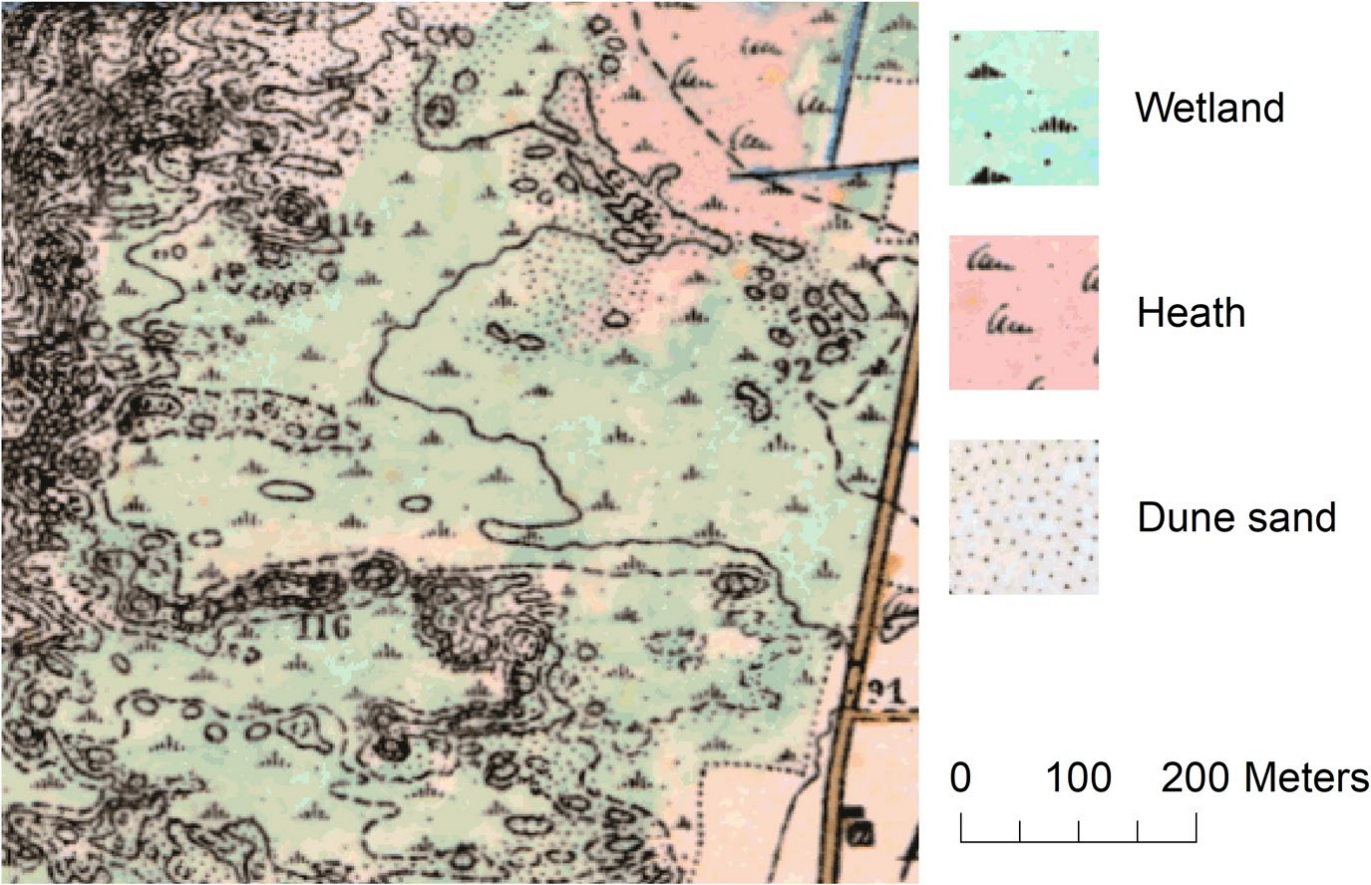
Example of combined signatures and colours for LULC categories in the HMB from the Hirtshals study area

Our results also show transitions between different categories of open nature especially in the Hirtshals study area. Here, approximately 50% of the 1880 dune sand area changed to heath or dry grassland. This might be interpreted as an actual change from open sand to grass, herbaceous, and/or scrub vegetation due to active prevention of sand drift and due to natural succession. Particularly since the second half of the nineteenth century, active prevention of sand drift included establishment of conifer plantations as well as planting of marram grass and dispersion of heath vegetation (Ejrnæs et al., 2021; Skarregaard, 2008). However, this change could also be related to different categorisations of dune sand between the historical and contemporary data. The survey instructions for the HMB do not provide information on thresholds for the categorisation of vegetation and soil condition. In fact, in some cases, colours and signatures for different LULC categories in the HMB are represented in combination, e.g. the heath colouration and symbology in combination with wetland and/or sand symbology (see Fig. 9). This indicates that such combination was used to represent fuzzy borders in areas with a complex composition between different classes.

In general, the HMB denote a boundary of wetland, heath, and dune sand to each other, to other mapped categories and to other parts of the maps as a faint pecked line; locally the applied colour wash has not respected these lines. It was therefore considered to use these boundary lines as an assist in the mapping, but signature variability and overlap to other HMB lines prevented that, given the available resources.

Our study has demonstrated the importance of including manual examination of map-related source material such as the survey instructions in making machine-readable LULC category layers from historical topographic maps. Survey instruction content was key in assisting the understanding of the HMB mapping of wetland but could provide only limited input for interpretation of cases where the HMB indicate combinations of categories, as have been discussed above. This was to some degree remedied by historical analysis of the technical and conceptual development in military survey in the decades leading up to the final legend for the HMB map (Svenningsen et al., 2022). However, an important observation from our study is that there can remain at least the possibility for knowledge gaps in confidently knowing why a category marking has been placed where it has in a historical map. Here, additional historical information such as early photography or landscape paintings could potentially be a source for comparing mapping with actual situation on the ground (Lacina & Halas, 2015; Skokanová et al., 2021). Specialised knowledge of the processes associated with the Danish shifting sand areas could have also assisted in this. However, such issues do not only apply to historical maps. An underlying point here is that landscape realities, such as ones associated with natural or semi-natural LULC categories, are challenging to represent as map geodata, also in modern mapping (Yang et al., 2017).

We demonstrate the potential of applying automated methods, which will allow for cost-effective un-locking of historical maps from larger areas or even complete countries or regions for multiple LULC categories. There have been key advances in Internet-based work since the historical map digitisation part of this study was undertaken in 2017–2018, including those related to increased workplace use of the Internet during the COVID pandemic (Mouratidis & Papagiannakis, 2021). That has included increasing use of crowd-sourcing Internet methods (Novak & Ostash, 2022; Sobotkova et al., 2023). For scaling-up from a study such as ours, crowd-sourced methods could be usefully applied, in particular for the generation of reference data for accuracy assessment, with assignment of points to categories by multiple participants providing key insight into ambiguities in the source map material.

In general, methods for automated extraction of machine-readable layers from historical topographic maps have also advanced since this study was undertaken, including key recent advances in artificial intelligence (Garcia-Molsosa et al., 2021; Wong et al., 2022). Future work with the HMB should consider further method developments based on these advances. At the same time, we believe that several aspects of the methods we have applied have enduring relevance; these include the use of simple image data derivative image layers combined with layer thresholding and the power of simple object-based contextual rules and procedures for going from an initial digitisation to a higher quality final product.

Despite advances in digital map processing, the modular approach we applied in our study is still valid, since, particularly when accounting for the complexity of historical topographic maps, one algorithm or method can most probably not be applied to all LULC categories. Furthermore, the interdisciplinary approach we applied is still central, since an understanding of the definition and cartographic depiction of LULC categories in applied historical maps is pivotal to which methods for automated extraction to apply.

Only a sub-part of the total information within the HMB was extracted in our study. Several other pieces of information could have potentially been extracted but the need to focus upon the five selected target categories precluded that. Changes in settlement over 100 + years are relevant as elements and drivers of landscape dynamics (Allan et al., 2022). Individual buildings were surveyed and mapped in the HMB and comparison to reference data, such as modern buildings, indicates a high level of building footprint accuracy in the HMB. In a similar LULC context, the HMB represented networks of transportation routes, such as roads could probably also be extracted without major further method development. Furthermore, for future assessment, adding additional historical maps, such as the Lave Målebordsblade, which were surveyed in the period from 1901 to 1970, would give more insight into spatio-temporal LULC dynamics.

## Conclusion

Historical topographic maps serve as potent resources for discerning past landscape patterns, particularly as they stand as highly dependable depictions of landscapes predating the advent of aerial photography and satellite imagery. Yet, maps, also if scanned and geo-rectified, are merely pictures. To integrate information from historical maps into a GIS, it becomes imperative to extract and comprehend the information encapsulated within them through map processing techniques. In contrast to laborious visual interpretation and manual delineation, digital map processing encompasses computational procedures designed to automatically or semi-automatically generate digital spatial datasets representing geographic features depicted in maps.

In two study areas, representing typical Danish landscapes, we successfully employed OBIA, vector GIS, CIS, and ML processes to generate machine-readable LULC layers from late nineteenth-century topographic maps. An accuracy assessment applied to the extracted LULC categories indicated an achieved overall accuracy of 92.3%.

Comparison between our mapping from late nineteenth-century topographic maps with a contemporary map revealed landscape dynamics characterised by a reduction in heath, wetland, and dune sand due to cultivation and afforestation. Nevertheless, dune sand also exhibited a transition to heath and dry grassland. We conclude that the automated production of machine-readable LULC categories from historical maps provides a less time-consuming and more resource-efficient alternative to manual vectorisation. Our findings also underscore the importance of understanding mapped LULC categories in both historical and contemporary maps for interpreting LULC dynamics.

## Supplementary Information

Below is the link to the electronic supplementary material.

Supplementary file1 (JPG 3144 KB)

Supplementary file2 (DOCX 33 KB)

## Data Availability

The datasets generated and analysed during the current study are available at the homepage of the department of Environmental Science, Aarhus University at https://envs.au.dk/en/research-areas/society-environment-and-resources/land-use-and-gis/land-cover-and-land-use-in-the-late-1800s-for-download. Applied topographical maps from the Agency for Data Supply and Infrastructure are available at https://dataforsyningen.dk/data/3577. Scanned and geo-rectified map sheets of the HMB maps at the Royal Library are available at https://loar.kb.dk/collections/25dabe73-e917-49a2-ac42-3dbb21a99474.
