# Supplementary figures and images for: Assessing spatially explicit long-term landscape dynamics based on automated production of land category layers from Danish late nineteenth-century topographic maps in comparison with contemporary maps
Source: Environ Monit Assess. 2025 Jan 25;197(2):195. doi: 10.1007/s10661-025-13634-1 (PMC11761518; doi:10.1007/s10661-025-13634-1)

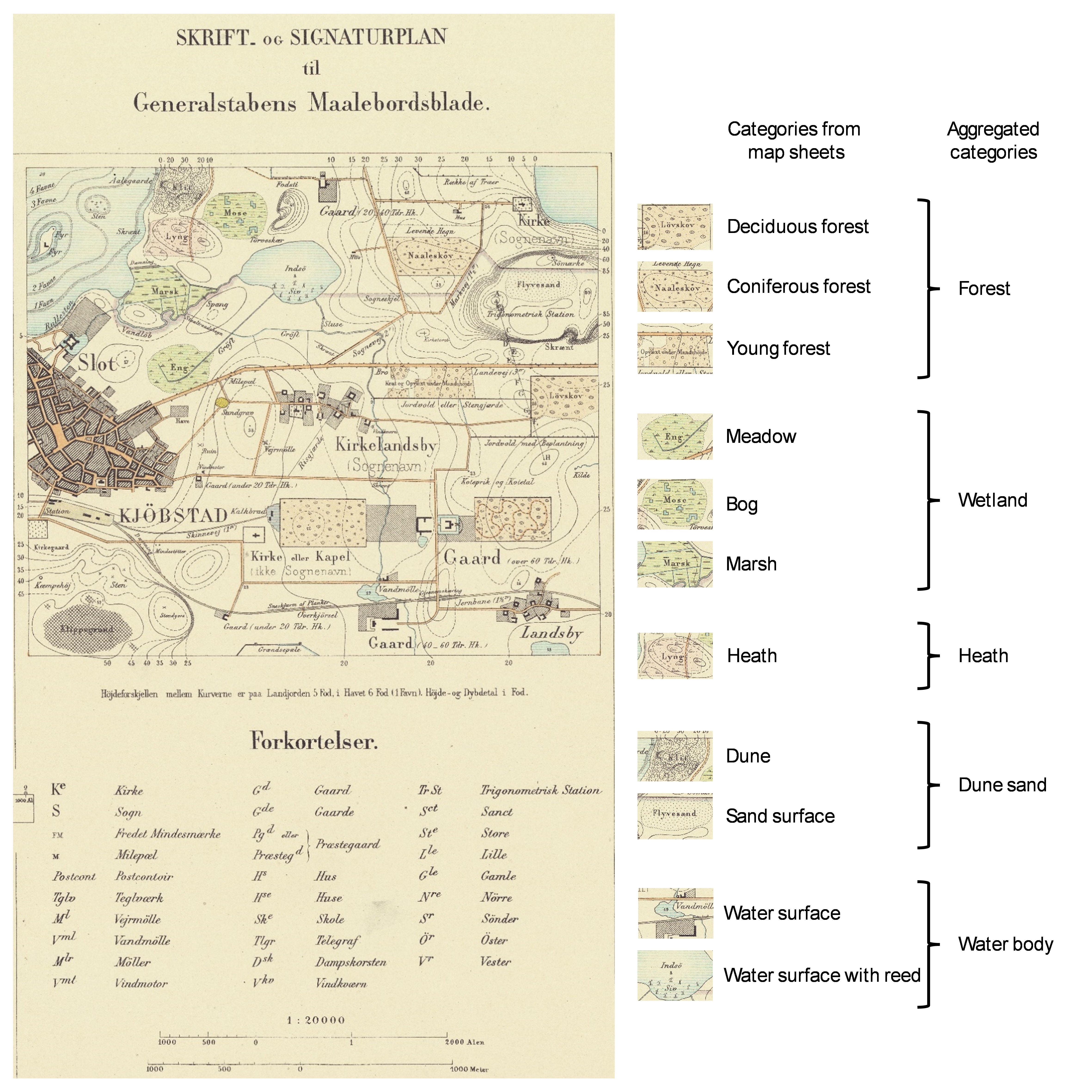

Supplement: Supplementary file 1 — Supplementary file1 (JPG 3144 KB) [file 10661_2025_13634_MOESM1_ESM.jpg]
