# Supplementary material for: Assessing spatially explicit long-term landscape dynamics based on automated production of land category layers from Danish late nineteenth-century topographic maps in comparison with contemporary maps
Source: Environ Monit Assess. 2025 Jan 25;197(2):195. doi: 10.1007/s10661-025-13634-1 (PMC11761518; doi:10.1007/s10661-025-13634-1)
Supplement: Supplementary file 2 — Supplementary file2 (DOCX 33 KB) [file 10661_2025_13634_MOESM2_ESM.docx]

Table S1: Aggregation of LULC categories from Basemap03

| Basemap Object Code | Basemap Object Name | Aggregated code | Aggregated Name |
| --- | --- | --- | --- |
| 20110200 | Lake | 3000 | Freshwater |
| 20201100 | Public area | 4000 | Built-up |
| 20310100 | Wet meadow | 2010 | Wetland |
| 20310200 | Coastal swamp | 2010 | Wetland |
| 20310300 | Mire / bog | 2010 | Wetland |
| 20320100 | Heather | 2020 | Heath |
| 20320400 | Plain | 2021 | Dry grassland |
| 20320500 | Dry meadow | 2021 | Dry grassland |
| 20320600 | Dune | 2030 | Dune sand |
| 20320800 | Beach | 2030 | Dune sand |
| 20600200 | Uncultivated area | 2021 | Dry grassland |
| 20600300 | Hillside | 2021 | Dry grassland |
| 20610300 | Ash | 1000 | Forest |
| 20610400 | Aspen | 1000 | Forest |
| 20610600 | Birch | 1000 | Forest |
| 20610700 | Beech | 1000 | Forest |
| 20610900 | Contorta | 1000 | Forest |
| 20611000 | Oak | 1000 | Forest |
| 20611100 | Alder | 1000 | Forest |
| 20612000 | Lime | 1000 | Forest |
| 20612100 | Norway maple | 1000 | Forest |
| 20612800 | Scrub | 1000 | Forest |
| 20620200 | Grandis | 1000 | Forest |
| 20620300 | Not specified coniferous tree | 1000 | Forest |
| 20620500 | Mountain pine | 1000 | Forest |
| 20620700 | Cypress | 1000 | Forest |
| 20620800 | Douglas fir | 1000 | Forest |
| 20621000 | Frensh mountain pine | 1000 | Forest |
| 20621100 | Common silver fir | 1000 | Forest |
| 20621400 | Larch | 1000 | Forest |
| 20621500 | Norman spruce | 1000 | Forest |
| 20621600 | Nobilis | 1000 | Forest |
| 20621700 | Omorika | 1000 | Forest |
| 20621800 | Austran pine | 1000 | Forest |
| 20621900 | Common spruce | 1000 | Forest |
| 20622000 | Sita spruce | 1000 | Forest |
| 20622100 | Scotsh pine | 1000 | Forest |
| 20622300 | Hemlock | 1000 | Forest |
| 20622400 | White spruce | 1000 | Forest |
| 20700100 | Field | 5010 | Agriculture |
| 30000100 | Wet meadow | 2010 | Wetland |
| 30000200 | Heather | 2020 | Heath |
| 30000300 | Mire / bog | 2010 | Wetland |
| 30000400 | Dry meadow | 2021 | Dry grassland |
| 30000500 | Coastal meadow | 2010 | Wetland |
| 30000600 | Lake | 3000 | Freshwater |
| 40212000 | Shifting dunes along the shoreline with Ammophila  arenaria (white dunes) | 2030 | Dune sand |
| 40213000 | Fixed coastal dunes with herbaceous vegetation (grey dunes) | 2021 | Dry grassland |
| 40216000 | Dunes with Hippophae rhamnoides | 1000 | Forest |
| 40219000 | Humid dune slacks | 2010 | Wetland |
| 40225000 | Coastal dunes with Juniperus spp. | 1000 | Forest |
| 40403000 | European dry heaths | 2020 | Heath |
| 40513000 | Juniperus communis formations on heaths or calcareous grasslands | 1000 | Forest |
| 40623000 | Species-rich Nardus grasslands, on siliceous substrates in mountain areas (and submountain areas, in Continental Europe) | 2021 | Dry grassland |
| 40641000 | Molinia meadows on calcareous, peaty or clayey-silt laden soils (Molinion caeruleae) | 2010 | Wetland |
| 40714000 | Transition mires and quaking bogs | 2010 | Wetland |
| 40722000 | Petrifying springs with tufa formation (Cratoneurion) | 2010 | Wetland |
| 40723000 | Alkaline fens | 2010 | Wetland |
| 40910100 | Bog woodland | 1000 | Forest |
| 40910200 | Alluvial forests with Alnus glutinosa and Fraxinus excelsior (Alno-Padion, Alnion incanae, Salicion albae) | 1000 | Forest |
| 40911000 | Luzulo-Fagetum beech forests | 1000 | Forest |
| 40916000 | Sub-Atlantic and medio-European oak or oakhornbeam forests of the Carpinion betuli | 1000 | Forest |
| 40919000 | Old acidophilous oak woods with Quercus robur on sandy plains | 1000 | Forest |
| 50311900 | Recreation area | 4000 | Built-up |
| 50600000 | Land | 9000 | Other |
| 50700000 | Sea | 3010 | Sea |
| 50990103 | Technical area, Energy supply plant | 4000 | Built-up |
| 50990104 | Technical area, Solar power | 4000 | Built-up |
| 50990106 | Technical area, Train station/rail area | 4000 | Built-up |
| 50990109 | Technical area, Recreation area | 4000 | Built-up |
| 50990110 | Technical area, Sports ground | 4000 | Built-up |
| 50990116 | Technical area, Car park | 4000 | Built-up |
| 50990118 | Technical area, Unknown | 4000 | Built-up |
| 50990118 | Technical area, Unknown | 4000 | Built-up |
| 50990201 | Basin, Other | 4000 | Built-up |
| 50990204 | Basin, Wastewater treatment plant | 4000 | Built-up |
| 50990205 | Basin, Swimming pool | 4000 | Built-up |
| 50990206 | Basin, Unknown | 4000 | Built-up |
| 50991700 | Forest | 1000 | Forest |
| 50991800 | Heather | 2020 | Heath |
| 50991900 | Wetland | 2010 | Wetland |
| 50992100 | Sand / dune | 2030 | Dune sand |
| 50992200 | Resource extraction | 4000 | Built-up |
| 50994201 | Lake, Fishpond | 3000 | Freshwater |
| 50994202 | Lake, Lake | 3000 | Freshwater |
| 50994400 | Harbour | 4000 | Built-up |
| 50994601 | Building, Building | 4000 | Built-up |
| 50994602 | Building, Tank/silo | 4000 | Built-up |
| 50994604 | Building, Greenhouse | 4000 | Built-up |
| 50994605 | Building, Solar panel | 4000 | Built-up |
| 50995200 | City centre | 4000 | Built-up |
| 50995300 | Business | 4000 | Built-up |
| 50995400 | Low built up | 4000 | Built-up |
| 50995500 | High built up | 4000 | Built-up |
| 50995701 | Edge of stream, >= 12 m width | 3000 | Freshwater |
| 50996301 | Road centreline, Other road, paved | 4000 | Built-up |
| 50996302 | Road centreline, Other road, not paved | 4000 | Built-up |
| 50996303 | Road centreline, Other road, ND | 4000 | Built-up |
| 50996305 | Road centreline, Highway | 4000 | Built-up |
| 50996401 | Railway, Visible | 4000 | Built-up |
| 50996501 | Stream centreline, 2.5 - 12 m width | 4000 | Built-up |
| 50997001 | Runway, Take off/landing | 4000 | Built-up |
| 50997800 | Burial ground | 4000 | Built-up |
| 60000100 | Spring barley | 5010 | Agriculture |
| 60000300 | Oat | 5010 | Agriculture |
| 60001000 | Winter barley | 5010 | Agriculture |
| 60001100 | Winter wheat | 5010 | Agriculture |
| 60001500 | Hybrid rye | 5010 | Agriculture |
| 60001600 | Triticale | 5010 | Agriculture |
| 60014900 | Seed potato (certified) | 5010 | Agriculture |
| 60021000 | Spring barley, whole crop | 5010 | Agriculture |
| 60021600 | Silo maize | 5010 | Agriculture |
| 60024700 | Environmental grass (0 N), in rotation | 5010 | Agriculture |
| 60025200 | Permanent grass, normal yield | 5010 | Agriculture |
| 60026000 | Clover grass, <50% clover | 5010 | Agriculture |
| 60026300 | Grass without clover | 5010 | Agriculture |
| 60030800 | Environmental focus area with fallow for mowing | 5010 | Agriculture |
| 60031000 | Fallow for mowing | 5010 | Agriculture |
| 60058300 | Christmas tree, decorative greenery | 5010 | Agriculture |
| 60070100 | Green grain from spring barley | 5010 | Agriculture |
| 60070400 | Green grain from spring rye | 5010 | Agriculture |
| 61000000 | Field block, not classified | 5010 | Agriculture |
| 61000100 | Field block, forest | 1000 | Forest |
| 61000200 | Field block, periodical crop | 5010 | Agriculture |
| 61000300 | Field block, permanent crop | 5010 | Agriculture |
| 61000400 | Field block, extensive | 5010 | Agriculture |
| 70000200 | Spring wheat | 5010 | Agriculture |
| 70000400 | Other spring cereal | 5010 | Agriculture |
| 70000500 | Maize to maturity | 5010 | Agriculture |
| 70000700 | Cereal/pulse, max. 50% pulse | 5010 | Agriculture |
| 70000900 | Wither spelt | 5010 | Agriculture |
| 70001300 | Wither wheat, near cereal | 5010 | Agriculture |
| 70001400 | Winter rye | 5010 | Agriculture |
| 70002200 | Winter rape | 5010 | Agriculture |
| 70002400 | Sunflower | 5010 | Agriculture |
| 70003000 | Pea | 5010 | Agriculture |
| 70003100 | Broad bean | 5010 | Agriculture |
| 70004000 | Flax grown as an oilseed crop | 5010 | Agriculture |
| 70004200 | Hemp | 5010 | Agriculture |
| 70005600 | Spring triticale | 5010 | Agriculture |
| 70010100 | Rai grass seed | 5010 | Agriculture |
| 70010200 | Rai grass seed, fall planted | 5010 | Agriculture |
| 70010300 | Italian rai grass seed | 5010 | Agriculture |
| 70010900 | Festulolium | 5010 | Agriculture |
| 70011100 | Festuca littorea seed | 5010 | Agriculture |
| 70011600 | Rai grass, hybrid | 5010 | Agriculture |
| 70012000 | Clover seed | 5010 | Agriculture |
| 70012400 | Spinach seed | 5010 | Agriculture |
| 70012600 | Other seed for sowing | 5010 | Agriculture |
| 70015000 | Seed potato (own generation) | 5010 | Agriculture |
| 70015100 | Starch potato | 5010 | Agriculture |
| 70015200 | Potato for consumption | 5010 | Agriculture |
| 70015300 | Potato other | 5010 | Agriculture |
| 70017100 | Lucerne for harvest and own fodder | 5010 | Agriculture |
| 70017200 | Lucerne for harvest and own fodder, min. 25% grass | 5010 | Agriculture |
| 70017300 | Clover for harvest | 5010 | Agriculture |
| 70021200 | Oat, whole crop | 5010 | Agriculture |
| 70021300 | Dredge corn, spring planted | 5010 | Agriculture |
| 70021400 | Cereal, pulse, whole crop max. 50% pulse | 5010 | Agriculture |
| 70021500 | Pea, whole crop | 5010 | Agriculture |
| 70022200 | Winter rye, whole crop | 5010 | Agriculture |
| 70023000 | Spring cereal, green grain | 5010 | Agriculture |
| 70023400 | Cereal/pulse, green grain. max. 50% pulse | 5010 | Agriculture |
| 70024800 | Permanent grass at water drilling | 5010 | Agriculture |
| 70024900 | Cultivated grass at water drilling | 5010 | Agriculture |
| 70025000 | Permanent grass, very low yield | 5010 | Agriculture |
| 70025100 | Permanent grass, low yield | 5010 | Agriculture |
| 70025400 | Environmental grass (0 N) | 5010 | Agriculture |
| 70025500 | Permanent grass, <50% clover | 5010 | Agriculture |
| 70025600 | Permanent grass, >50% clover | 5010 | Agriculture |
| 70025700 | Permanent grass, no clover | 5010 | Agriculture |
| 70026100 | Clover grass, >50% clover | 5010 | Agriculture |
| 70026400 | Grass and clover grass without N-norm | 5010 | Agriculture |
| 70026600 | Grass <50% clover, extremely low yield | 5010 | Agriculture |
| 70026700 | Grass <50% clover, very low yield | 5010 | Agriculture |
| 70026800 | Grass <50% clover, low yield | 5010 | Agriculture |
| 70026900 | Turf | 5010 | Agriculture |
| 70027100 | Areas for recreation purposes | 5010 | Agriculture |
| 70027600 | Permanent grass/clover grass without N-norm, <50% clover | 5010 | Agriculture |
| 70028000 | Sugar cane, fodder | 5010 | Agriculture |
| 70028200 | Marrow-stem kale | 5010 | Agriculture |
| 70028400 | Grass with pulses, >50 % pulses | 5010 | Agriculture |
| 70028500 | Grass and clover without N-norm, >50 % clover (in rotation) | 5010 | Agriculture |
| 70028600 | Permanent grass and clover grass wihtout N-norm, >50 % clover | 5010 | Agriculture |
| 70030900 | Uncultivated area at water drilling | 5010 | Agriculture |
| 70031100 | Afforestation on former agricultural land | 1000 | Forest |
| 70031200 | 20 years set-aside | 5010 | Agriculture |
| 70031800 | Agri-envrionmental scheme, not agricultural land | 5010 | Agriculture |
| 70032100 | Environmental initiative, not agricultural land | 5010 | Agriculture |
| 70032300 | Environmental focus area at water drilling | 5010 | Agriculture |
| 70032500 | Environmental focus area with flower fallow | 5010 | Agriculture |
| 70032700 | Environmental focus area, fringe with summer mowing | 5010 | Agriculture |
| 70033800 | Fallow, spring mowing | 5010 | Agriculture |
| 70033900 | Environmental focus area, fallow, spring mowing | 5010 | Agriculture |
| 70040300 | Cauliflower | 5010 | Agriculture |
| 70040400 | Broccoli | 5010 | Agriculture |
| 70040700 | Carrot | 5010 | Agriculture |
| 70042300 | Sweet corn | 5010 | Agriculture |
| 70042400 | Peas for consumption | 5010 | Agriculture |
| 70042900 | Jerusalem artichoke for consumption | 5010 | Agriculture |
| 70043400 | Vegetable, other, outdoors | 5010 | Agriculture |
| 70045000 | Vegetable, other | 5010 | Agriculture |
| 70048700 | Agroforestry | 1000 | Forest |
| 70049600 | Medicine plants woody | 5010 | Agriculture |
| 70049700 | Nursery, woody plants for sale | 5010 | Agriculture |
| 70051300 | Strawberry | 5010 | Agriculture |
| 70051400 | Blackcurrant | 5010 | Agriculture |
| 70051700 | Blackberry | 5010 | Agriculture |
| 70051800 | Raspberry | 5010 | Agriculture |
| 70051900 | Blueberry | 5010 | Agriculture |
| 70052000 | Cherry without undergrowth | 5010 | Agriculture |
| 70052200 | Plum without undergrowth | 5010 | Agriculture |
| 70052400 | Sweet cherry without undergrowth | 5010 | Agriculture |
| 70052800 | Apple | 5010 | Agriculture |
| 70052900 | Pear | 5010 | Agriculture |
| 70053000 | Grape | 5010 | Agriculture |
| 70057800 | Afforestation- improvement of aquatic environment and groundwater protection | 1000 | Forest |
| 70058000 | Forestry, common | 1000 | Forest |
| 70058100 | Young plantation in forest with tree height under 3 m | 1000 | Forest |
| 70058500 | Christmas tree, decorative greenery on agricultural land | 5010 | Agriculture |
| 70058600 | Public afforestation | 1000 | Forest |
| 70058900 | Sustainable afforestation | 1000 | Forest |
| 70059200 | Willow | 5010 | Agriculture |
| 70059300 | Poplar | 5010 | Agriculture |
| 70059600 | Elephant grass | 5010 | Agriculture |
| 70060200 | Willow on environmental focus sites | 5010 | Agriculture |
| 70060300 | Poplar on environmental focus sites | 5010 | Agriculture |
| 70070200 | Green grain from spring wheat | 5010 | Agriculture |
| 70070300 | Green grain from spring oat | 5010 | Agriculture |
| 70070700 | Green grain from winter wheat | 5010 | Agriculture |
| 70070900 | Green grain from winter rye | 5010 | Agriculture |
| 70090300 | Open nature in protected forest | 5010 | Agriculture |
| 70090700 | Organic nature area | 5010 | Agriculture |
| 70090800 | Nature area, application for environmental subsidies | 5010 | Agriculture |
| 80000100 | Cadastre, road | 4000 | Built-up |
| 80000200 | Cadastre, rail | 4000 | Built-up |
